# Increased expression of TIGIT and KLRG1 correlates with impaired CD56^bright^ NK cell immunity in HPV16-related cervical intraepithelial neoplasia

**DOI:** 10.1186/s12985-022-01776-4

**Published:** 2022-04-12

**Authors:** You Nie, Dandan Liu, Wen Yang, Yazhuo Li, Lihua Zhang, Xia Cheng, Ruyu Chen, Bingbing Yuan, Guangzheng Zhang, Hongwei Wang

**Affiliations:** 1 Department of Pathology, Fourth Medical Centre of Chinese PLA (People’s Liberation Army) General Hospital, 51 Fucheng Road, Beijing, China; 2 Basic Medicine College, Zhengzhou University, 100 Science Avenue, Henan, China; 3 Department of Gynaecology and Obstetrics, Chinese PLA (People’s Liberation Army) General Hospital, Beijing, China

**Keywords:** Cervical intraepithelial neoplasia, Human papillomavirus 16, Natural killer cell, Cytokines, TIGIT-PVR, KLRG1-Cadherin

## Abstract

**Background:**

The onset and progression of cervical intraepithelial neoplasia (CIN) are closely associated with the persistent infection of high-risk HPV (especially type16), which is mainly caused by immune escape. Natural killer (NK) cells play an important role against virally infected cells and tumor cells through a fine balance of signals from multiple surface receptors. Overexpression of non-MHC-I specific inhibitory receptors TIGIT, KLRG1, Siglec-7, LAIR-1, and CD300a on NK cells correlates with cellular exhaustion and immune evasion, but these receptors have not been investigated in CIN. The aim of the present study was to examine the potential role of NK cell non-MHC-I specific inhibitory receptors expression in immune escape from HPV16(+)CIN patients.

**Methods:**

The subset distribution, IFN-γ and TNF-α expression levels and immunophenotype of TIGIT, KLRG1, Siglec-7, LAIR-1, and CD300a of NK cells were investigated in peripheral blood mononuclear cell samples by flow cytometry from 82 women who were HPV16(+) with CIN grades 0, I, II–III or HPV(−) CIN 0. Immunohistochemistry was applied to detect the expression of ligands for NK receptors in the cervical tissues. HPV types were identified by PCR assays.

**Results:**

The HPV16(+) subjects with high-grade lesions had an increased number of circulating peripheral blood CD56^bright^ NK cells with reduced functionality and IFN-γ secretion. The expression levels of the inhibitory molecules TIGIT and KLRG1 on CD56^bright^ NK cells increased in parallel with increasing CIN grade. In addition, TIGIT and KLRG1 related ligands, Poliovirus receptor (PVR), N-Cadherin and E-Cadherin expression level was also elevated with increasing CIN grade.

**Conclusions:**

Our results suggest that up-regulation of the inhibitory TIGIT, KLRG1 and their ligands may negatively regulate cervical CD56^bright^ NK-mediated immunity to HPV16 and contribute to the progression of CIN. These results may facilitate the development of early-warning immune predictors and therapeutic strategies for HPV16(+) CIN based on the TIGIT and KLRG1 inhibitory pathways of NK cells.

## Introduction

Despite the existence of several effective preventive vaccines [1], cervical cancer remains the most common malignancies of the female genital tract. According to recent GLOBOCAN statistics, there are approximately 604,000 new cases of cervical cancer and 342,000 deaths annually [2]. Persistent infection with high-risk subtype Human Papillomaviruses (HPV) [3, 4], especially HPV16, is a precursor of precancerous lesions of the cervix, also known as cervical intraepithelial neoplasia (CIN), which eventually leads to cancer in many cases [5, 6]. Natural killer (NK) cells are an important component of innate immunity and represent the first line of defense against viral infections. Receptors, activators or inhibitors, expressed on the surface of NK cells can contribute to the NK cell activation [7]. Research has shown that NK cells appear in early HPV-infected lesions, can be essential to the pathogenesis of the lesions [8]. Most HPV infections are transient and self-limiting, nevertheless, in approximately 1–2% of the affected it might persist, suggesting that HPV may have evolved an escape strategy in response to the pursuit of NK cells by regulating its activation [9, 10]. Currently, the drivers for HPV to escape NK cell immune response in the occurrence and progression of CIN and cervical cancer are still unclear, especially the action mechanism of different NK cell subsets in the progression of HPV-related CIN.

In recent years, with the development of immune checkpoint blockade treatment, NK cells have shown promise for targeted therapy [11]. Previous studies have demonstrated that NK cells play a pivotal role in the immune response against early-stage HPV-associated CIN, especially in low-grade CIN tissues with a relatively low viral load. Besides, NK cells, especially the CD56^bright^NK cells with immunomodulatory properties, can infiltrate and attack cancerous tissues more easily. Unlike B cells and T cells, the activation of NK cells relies heavily on the recognition of major histocompatibility complex (MHC)-I-like molecules by inhibitory receptors to distinguish between “self” and “non-self” [7]. In fact, many virus-infected cells and cancer cells down-regulate the expression of MHC-I molecules to escape the detection of cytotoxic CD8^+^T cells. Remarkably, NK cells can recognize and respond to those target cells with “missing-self” phenotype and attack them [7, 12–14]. However, despite the loss expression of MHC-I molecules, NK cells still show reduced function in patients with CIN and cervical cancer [15]. Based on this, we believe that this may be associated with the non-MHC-1 molecular pathway described by previous researchers [16]. TIGIT, KLRG1, Siglec-7, LAIR-1, and CD300a are important non-MHC-I inhibitory receptors on NK cells, involved in the NK cell-mediated regulation of various diseases [17–21]. However, it remains unclear whether non-MHC-I inhibitory receptors are expressed in HPV16(+) CIN, or whether the activation of related pathways suppresses NK cell function and is associated with impaired viral clearance.

To assess the roles of NK cells and these inhibitory receptors in the immunopathogenesis of HPV16(+) CIN, we examined the subset distribution, Interferon-γ (IFN-γ) and Tumor necrosis factor-α (TNF-α) expression levels and inhibitory immunophenotype expression levels of peripheral blood NK cells from HPV16(+) women with different CIN grades or HPV(−) women. We also measured levels of ligands corresponding to these inhibitory receptors in tissues of cervical. We found that the upregulation expression of TIGIT, KLRG1 on CD56^bright^NK cells and its ligands Poliovirus receptor (PVR), E-Cadherin, N-Cadherin is directly associated with HPV16(+) CIN, suggesting that NK cell surface inhibitory receptors may be the key components responsible for the compromised viral clearance due to impaired immunity in HPV16(+) subjects with CIN.

## Materials and methods

### Study participants and procedures

All the participants were recruited from the Fourth Medical Center of the Chinese PLA (People’s Liberation Army) General Hospital, Beijing, China. HPV samples and biopsy specimens were obtained during the second colposcopy for female subjects whose liquid-based cytology results during the first visit returned positive for atypical squamous cells, low-grade squamous intraepithelial lesion, or high-grade squamous intraepithelial lesion. These specimens were subjected to independent histological analysis by three pathologists and categorized as no intraepithelial neoplasia (CIN 0), low-grade intraepithelial neoplasia (CIN I), and high-grade intraepithelial neoplasia (CIN II–III). The exclusion criteria were as follows: pregnancy, other infectious diseases, acute reproductive tract infections, receiving anti-inflammatory therapy, tumors, immunodeficiency, and receiving immunosuppressive agents. The final cohort of 82 participants was divided into four groups: (1) 26 HPV16 subjects with CIN 0; (2) 17 HPV16(+) subjects with CIN 0; (3) 16 HPV16(+) subjects with CIN I; (4) 23 HPV16(+) subjects with CIN II–III. In addition, archival paraffin-embedded tissue samples were also collected and categorized into the above-mentioned four groups, each of which comprised 10 cases. All participants recruited in this study were aged between 20 and 60 (37.68 ± 10.36 years) and had signed an informed consent form. This study was reviewed and approved by the hospital ethics committee: Ethics Committee of the Fourth Medical Center of PLA General Hospital (2021KY006-HS001).

### Polymerase chain reaction (PCR) for HPV DNA typing

Cervical cytobrushes used to collect cervical exfoliated cells were placed into 10 ml gigenespecimen transport medium (Qiagen) and DNA was extracted from the cervical samples using the DNeasy tissue kit (Qiagen), according to the manufacturer’s instructions. The DNA was then subjected to qualitative PCR using specific primers. The β-Globin was amplified to verify that the samples contained DNA of sufficient quality and quantity for the HPV test. Water was used as a negative control in each PCR experiment.

### Flow cytometry

Blood samples (100 µl) collected from the participants were incubated for 25 min at 25 °C in a flow cytometry tube with the following specific antibodies (5 µl each): CD3 (563219, BD), CD16 (302018, Biolegend), CD56 (555518, BD), TIGIT (12-9500-42, eBioscience), DNAM-1 (559788, BD), NKG2A (130-113-565, Miltenyi Biotec), LAIR-1 (ab27744, Abcam), Siglec-7 (ab200558, Abcam), and KLRG1 (25–9488-42, eBioscience). In addition, an isotype control was prepared for each sample. After incubation, the reaction mixture in each tube was mixed thoroughly with 2 ml of hemolysin. The samples were then washed for flow cytometry analysis (BD FACSCanto™ Flow Cytometer) within 4 h.

### Cytokine flow cytometry assay

3 μl of blood samples were collected from the participants and subjected to density gradient centrifugation to isolate peripheral blood mononuclear cells (PBMC). After being washed and resuspended, freshly isolated PBMCs were seeded at a density of 10^6^ cells/well and incubated with 100 ng/ml of phorbol myristate acetate (524400, sigma), 1 μg/ml of ionomycin (407951, sigma), and 10 μg/ml of brefeldin A (555029, BD) at 37 °C and 5% CO_2_ for 6 h. Subsequently, the stimulated cells were incubated with 5 μl of CD56, CD3, CD16, and isotype controls (as mentioned above) in the dark at 25 °C for 25 min for cell surface labeling. The cells were then fixed and permeabilized using Fixation/Permeabilization Solution kit (555028, BD) and incubated in the dark with 10 μl of anti-IFN-γ (552887, BD) and 8 μl of anti-TNF-α (562083, BD) at 4 °C for 30 min. After washing, the samples were loaded for flow cytometry assay.

### Immunohistochemical (IHC) assay

Cervical biopsy samples were routinely embedded in paraffin and sectioned at a thickness of 3 μm, followed by hematoxylin–eosin (HE) staining. The HE-stained tissue sections were then examined by three experienced pathologists. Prior to IHC staining, the tissue sections were dewaxed in xylene and rehydrated in graded alcohol. The tissue sections were incubated with the following antibodies: PVR (ab267389, diluted at a ratio of 1:300, Abcam), E-Cadherin and N-Cadherin (MAB0738 and MAB0571, respectively, diluted at a ratio of 1:200, MXB Biotechnologies), followed by IHC staining using the streptavidin-peroxidase conjugate method. Negative controls were prepared with non-immune isotype-specific sera used in place of primary antibodies, while previously positively-stained tissue sections for the labeled proteins were taken as positive controls.

### Statistical analysis

Statistical analysis was performed using GraphPad 8.0.1. Flow cytometry data were expressed as percentages and median fluorescence intensity (MFI). A nonparametric test (Kruskal–Wallis test) was used for median comparisons and completed with Dunn’s multiple comparisons tests. In experiments involving histology or IHC, the figures shown are representative of the tissue sections. The Allred scoring system was used for IHC staining quantification [22]. Proportion scores were: 0, none; 1, less than one percent; 2, one percent to one tenth; 3, one tenth to one third; 4. one third to two thirds; 5, Two thirds or more. The intensity scores were: 1 for weak, 2 for medium, and 3 for strong. We evaluated 10 high-power fields (× 400 magnification) in each sample. Added the average of the intensity score and the proportional score as the Allred total score (range = 0–8). The image was captured with an Olympus BX40F microscope (Olympus). All statistical assessments were two-sided, and statistical significance was defined as *P* < 0.05.

## Results

### Increased peripheral blood CD56^bright^ NK cells counts in HPV16(+) CIN II–III

**Fig. 1:**
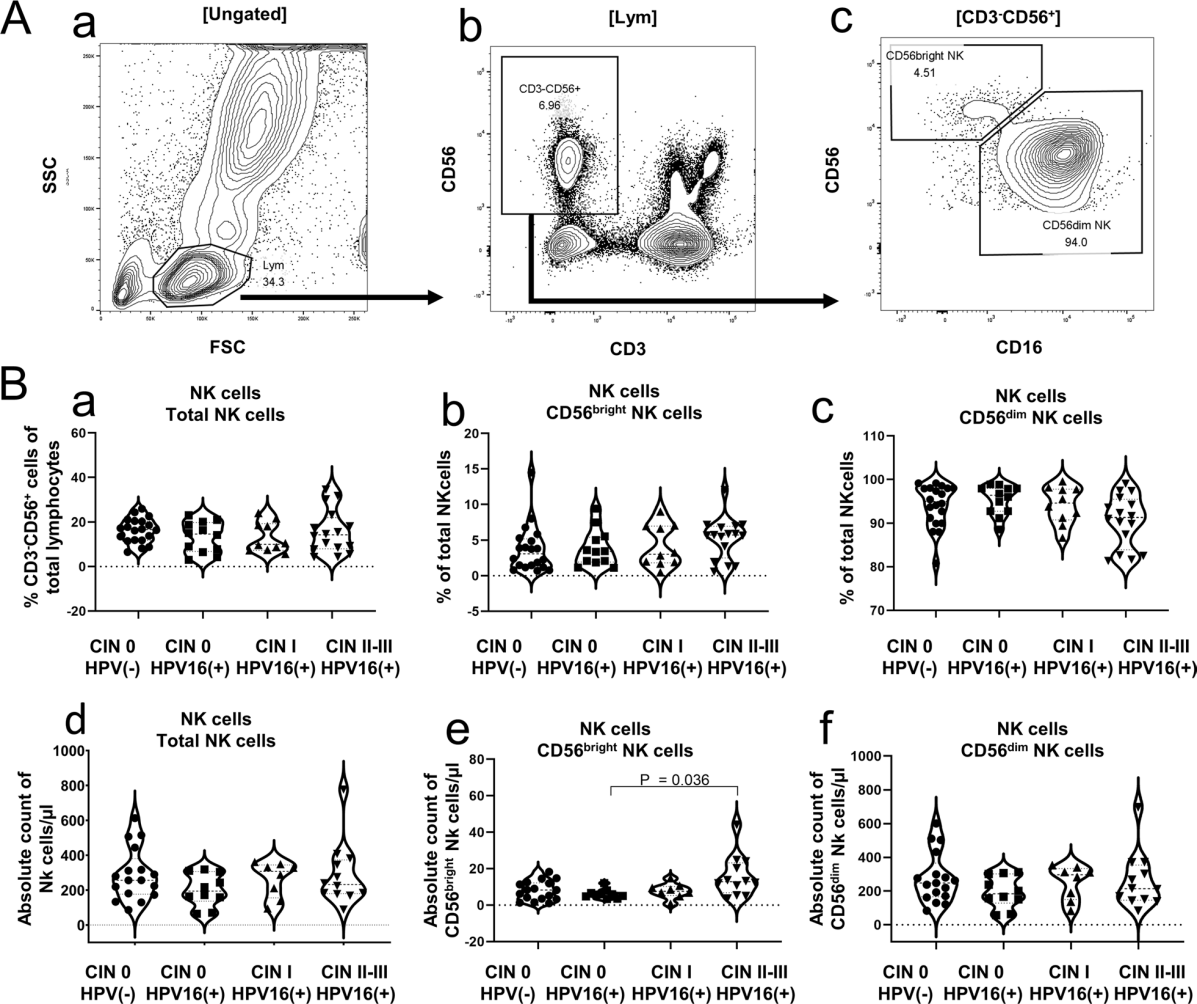
Increased number of circulating peripheral blood CD56^bright^ NK cells counts in HPV16(+) CIN II–III. **A**(a) Representative flow cytometry dot plots (SSC-A vs FSC-A). **A**(b) gated from **A**(a) and stained to obtain NK cells using CD3^−^ CD56^+^ as a marker. **A**(c) A representative dot plot in which two NK cell subsets were gated based on CD56 and CD16 expression: CD56^dim^ and CD56^bright^ NK cells. **B**(a–c) The percentage of NK cells and its subsets was analyzed from HPV16(+) women with different CIN grades or HPV(−) women using violin plots, in which each dot represents a donor. **B**(d–f) the absolute count of NK cells and their subsets was analyzed from HPV16(+) women with different CIN grades or HPV(−) women using violin plots, in which each dot represents a donor. The Kruskal–Wallis test was used to determine statistical significance

To probe the role of NK cell in the HPV16-infected CIN, the circulating NK cell (CD3^−^CD56^+^) subpopulations in the blood samples of the subjects from each group were analyzed by flow cytometry with appropriate gating strategies (Fig. 1A a–c). CD3^−^CD56^+^ is the most common phenotype of NK cells,. CD56^bright^ NK cells and CD56^dim^ NK cells are the common subtypes of CD3^−^CD56^+^ cells. We analyzed the distribution of total NK cells, CD56^bright^ NK cells, and CD56^dim^ NK cells in each group (Table 1, Fig. 1B a–c). The result showed that the CIN II–III group showed an increasing trend in the proportion (percentage relative to total NK cells) of circulating CD56^bright^ NK cells (*P* = 0.303)and a slightly lower proportion of circulating CD56^dim^ NK cells (*P* = 0.099) than the HPV(−) CIN 0 group and the HPV16(+) CIN I group, although these differences were not statistically significant. The number of cells per microliter of circulating blood for each NK cell subpopulation in each group was further calculated based on lymphocyte counts obtained via blood routine test. The CIN II–III group showed a significantly higher absolute count of CD56^bright^ NK cells than the HPV16(+) CIN 0 group (*P* = 0.036) (Table 1, Fig. 1B e), while there were no statistically significant differences in the absolute count of CD56^dim^ NK cells among the groups (Table 1, Fig. 1B f). Further, there were no significant differences in the percentage and absolute count of the total circulating NK cells among the groups (Table 1, Fig. 1B a, d). This finding suggests a potential role of CD56^bright^ NK cell for the HPV16(+) CIN progression, especially in the CIN II–III.

**Table 1:** Comparison between peripheral blood NK cell subsets in patients from the HPV16(+) CIN groups and the HPV(−) CIN 0 group

	CIN 0 HPV(−) (N = 20)		CIN 0 HPV16(+) (N = 11)		CIN I HPV16(+) (N = 10)		CIN II–III HPV16(+) (N = 16)		*P* value
	Median	IQR	Median	IQR	Median	IQR	Median	IQR	
Subset distribution									
CD3^−^CD56^+^NK cells (%)	16.69	11.85–20.47	14.66	6.84–19.81	10.04	7.38–19.37	14.33	8.04–22.02	0.555
CD3^−^CD56^+^NK cells (count)	256.6	177.0–380.4	194.6	136.7–305.8	307.4	155.5–344.1	232.2	179.3–373.3	0.407
CD56^bright^ NK cells (%)	3.090	1.258–5.195	3.420	1.918–5.423	3.030	1.830–6.990	5.730	2.518–6.965	0.303
CD56^bright^ NK cells (count)	8.301	3.409–12.23	5.864	4.499–6.948	7.630	5.318–10.27	12.84	6.793–23.21	**0.036**
CD56^dim^ NK cells (%)	94.95	90.31–98.02	96.38	92.70–97.94	94.60	90.78–97.79	91.33	83.90–95.45	0.099
CD56^dim^ NK cells (count)	248.0	166.6–365.8	183.9	129.1–302.4	296.1	149.5–333.0	214.1	146.6–354.2	0.623
CD56^dim^ NK cells									
TIGIT^+^ (%)	83.64	79.99–88.62	80.78	70.81–88.13	77.24	70.16–82.53	80.07	75.37–88.53	0.241
TIGIT^+^ (MFI)	2388	1873–3252	2122	1306–3254	1685	1399–2301	2080	1746–3246	0.234
CD226^+^ (%)	85.50	82.95–91.89	90.20	84.59–93.78	84.65	71.54–91.45	90.19	77.74–93.78	0.628
CD226^+^ (MFI)	1447	1364–1591	1596	1171–1739	1381	825.5–1475	1617	1210–1760	0.349
NKG2A^+^ (%)	29.46	15.88–47.20	38.91	33.96–48.97	34.64	27.29–43.55	29.83	23.68–42.14	0.684
NKG2A^+^ (MFI)	174.0	161.1–459.2	234.9	206.9–518.9	204.9	179.8–281.7	197.0	166.2–1074	0.406
CD300a^+^ (%)	98.74	94.87–99.29	98.32	97.36–98.98	98.33	97.04–98.75	97.87	96.67–98.86	0.804
CD300a^+^ (MFI)	2927	2087–3686	2733	1428–3403	3476	2788–4333	2688	1955–3923	0.366
LAIR-1^+^ (%)	98.85	96.27–99.52	99.41	98.44–99.79	98.26	97.34–98.87	98.39	96.31–99.28	0.137
LAIR-1^+^ (MFI)	2086	1321–2665	2798	2283–3064	1615	1442–2464	2012	1436–2392	0.256
Siglec7^+^ (%)	83.47	72.28–93.31	79.49	74.96–89.36	76.54	59.39–87.95	79.61	65.22–92.51	0.624
Siglec7^+^ (MFI)	11,164	7120–16,912	8153	4350–13,496	6774	2256–10,904	10,199	4249–19,750	0.280
KLRG-1^+^ (%)	24.40	20.63–38.53	25.73	16.89–41.76	45.69	31.26–53.97	40.38	33.43–52.90	**0.006**
KLRG-1^+^ (MFI)	214.0	143.9–487.8	310.6	222.5–603.3	328.1	161.8–738.5	498.5	262.3–1020	0.122
CD56^bright^ NK cells									
TIGIT^+^ (%)	23.02	15.12–33.16	28.72	24.55–34.03	26.34	17.79–32.73	35.24	29.63–38.51	**0.019**
TIGIT^+^ (MFI)	150.9	125.9–199.0	164.0	132.6–194.3	156.0	119.2–228.6	199.8	173.3–221.3	0.114
CD226^+^ (%)	96.29	94.83–98.12	97.10	93.95–97.67	97.48	76.04–98.87	96.10	93.19–97.63	0.742
CD226^+^ (MFI)	1918	1729–2218	2155	1370–2243	1717	768.1–2618	2085	1756–2324	0.916
NKG2A^+^ (%)	87.67	82.71–91.40	93.06	84.70–95.57	92.51	89.78–95.90	92.81	86.77–96.00	0.057
NKG2A^+^ (MFI)	5289	4741–7657	6854	5260–9279	6817	5103–9407	6764	4965–7938	0.291
CD300a^+^ (%)	99.57	98.49–100.0	99.56	98.62–99.94	99.21	98.69–99.69	100.0	99.03–100.0	0.362
CD300a^+^ (MFI)	7325	5285–8500	5945	4488–9307	9753	6058–12,490	8092	5945–10,576	0.199
LAIR-1^+^ (%)	99.45	97.89–99.74	99.51	99.03–99.87	99.21	97.66–99.76	99.61	98.97–100.0	0.458
LAIR-1^+^ (MFI)	2811	2434–3273	3236	2791–3737	2931	2415–3406	2692	2434–3078	0.450
Siglec7^+^ (%)	92.01	90.60–94.46	92.80	89.47–95.50	90.74	87.22–92.72	93.65	90.39–96.00	0.205
Siglec7^+^ (MFI)	5073	4134–6173	5683	4023–6452	5018	3910–5997	5782	3767–6822	0.842
KLRG-1^+^ (%)	2.470	0.745–6.345	9.710	6.703–40.37	20.64	17.87–28.00	4.240	1.560–8.998	** < 0.0001**
KLRG-1^+^ (MFI)	95.20	71.96–143.6	155.3	104.7–415.3	161.9	124.4–222.1	121.6	101.7–162.1	**0.021**

The distribution of circulating NK cell subsets and their immunophenotype was compared among the patients of HPV16(−) CIN 0 (N = 20), HPV16(+) CIN 0 (N = 11), HPV16(+) CIN I (N = 10) and HPV16(+) CIN II–III (N = 16). Kruskal–Wallis test was used to determine statistical significance. *P* values ≤ 0.05 were considered statistically significant and are indicated in bold

### Decreased peripheral blood CD56^bright^ NK cells function in HPV16(+) CIN II–III

**Fig. 2:**
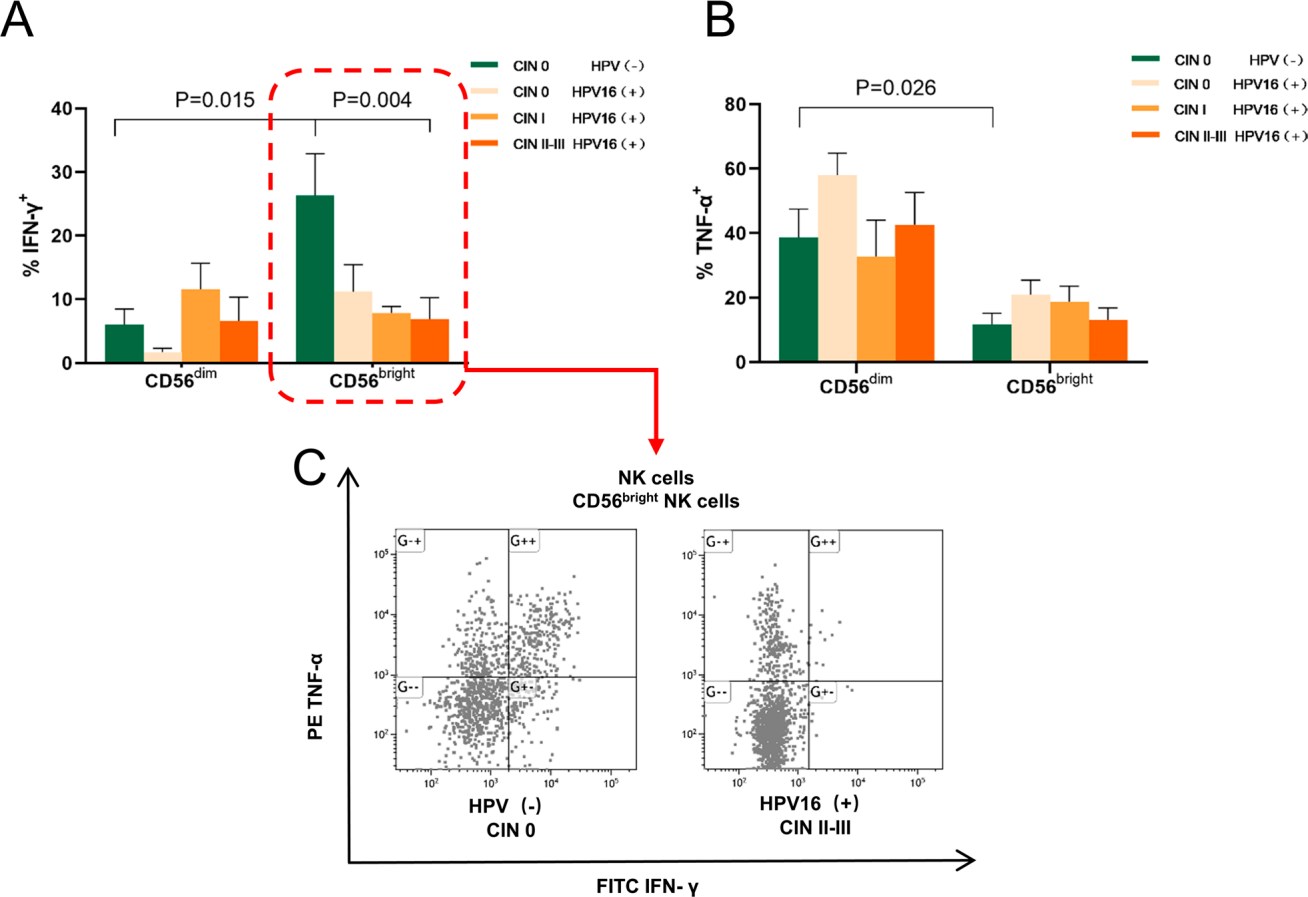
Circulating CD56^bright^ NK cells from HPV16(+) subjects with CIN showed a reduced ability to secrete IFN-γ. **A** Statistical graph displays percentage of IFN-γ created by CD56^dim^ and CD56^bright^ NK cells in the HPV16(+) women with different CIN grades and HPV(−) women. **B** Statistical graph displays percentage of TNF-α created by CD56^dim^ and CD56^bright^ NK cells in the HPV16(+) women with different CIN grades and HPV(−) women. **C** Representative dot plots of IFN-γ and TNF-α created by CD56^bright^ NK cells in the HPV(−) CIN 0 group and HPV16(+) CIN II–III groups

The expression of immunomodulatory cytokines, such as IFN-γ and TNF-α, is believed to affect persistent HPV infection and CIN progression. CD56^bright^ NK cell mainly characterized by cytokine secretion for defensing viral infections. We further examined the ability of peripheral blood NK cell subpopulations to secret IFN-γ and TNF-α via flow cytometry to explore the function of NK cells (especially CD56^bright^ subsets). The results showed that the CD56^bright^ NK cell subpopulation had a significantly higher secretion level of IFN-γ than the CD56^dim^ NK cell subpopulation in the HPV(−) CIN 0 (control) group (*P* = 0.015), while the CD56^dim^ NK cell subpopulation had a significantly higher secretion level of TNF-α than the CD56^bright^ NK cell subpopulation (*P* = 0.026) (Fig. 2A, B). However, the secretion level of IFN-γ by CD56^bright^ NK cells decreased with increasing CIN grade (Fig. 2A, C). Despite an increase in absolute count, the CD56^bright^ NK cells in the HPV16(+) CIN II–III group displayed significantly lower secretion of IFN-γ than that of the HPV(−) CIN 0 group (*P* = 0.004) (Fig. 2A, C). There were no significant differences in the secretion level of IFN-γ and TNF-α by CD56^dim^ NK cells between groups (Fig. 2B). These results indicate that the effector function of CD56^bright^ NK cells is reduced in patients with HPV16(+) CIN.

### Increased TIGIT and KLRG1 expression on CD56^bright^ NK cells in HPV16(+) CIN

**Fig. 3:**
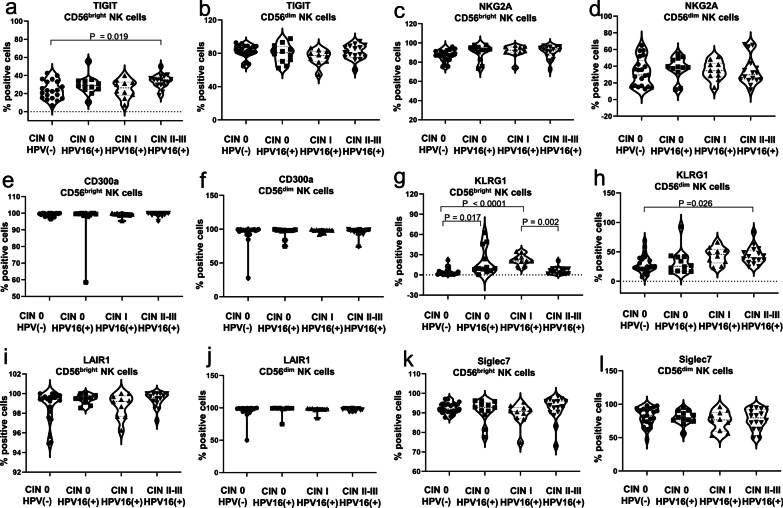
Increased expression of TIGIT and KLRG1 expression on CD56^bright^ NK cells in HPV16(+)-related CIN. **a**–**l** The percentage and MFI of inhibitory receptors TIGIT, NKG2A, CD300a, KLRG1, LAIR1, and Siglec-7 on NK cells were analyzed from HPV16(+) women with different CIN grades or HPV(−) women.. The Kruskal–Wallis test was used to test for statistical significance

The inhibitory receptors, TIGIT, NKG2A, CD300a, KLRG1, LAIR1, and Siglec-7, are responsible for the activation of NK cells. We further examined the expression of these inhibitory receptors on circulating peripheral blood NK cells in samples of different groups to explain why CD56^bright^ NK cells indicated reduced function in group of HPV16(+) CIN II–III. The results showed that the HPV16(+) CIN II–III group had a significantly higher percentage of TIGIT-positive CD56^bright^ NK cells than the HPV (−) CIN 0 group (*P* = 0.019) (Fig. 3a). However, there was no significant difference in the MFI of TIGIT on CD56^bright^ NK cells between the HPV (−) CIN 0 group and the HPV16(+) CIN groups (*P* = 0.114) (Table 1). Both the HPV16(+) CIN 0 group (*P* = 0.017) and the CIN I group (*P* < 0.0001) displayed a significantly higher percentage of KLRG1-positive CD56^bright^ NK cells than the HPV(−) CIN 0 group (Fig. 3g). However, the CIN II–III group had a lower percentage of KLRG1-positive CD56^bright^ NK cells than the CIN I group (*P* = 0.002) (Fig. 3g). Similarly, we observed a comparable differential distribution of the MFI of KLRG1 on CD56^bright^ NK cells between different groups (*P* = 0.021) (Table 1). In addition, we observed that the CIN II–III group had a significantly higher expression level of KLRG1 on CD56^dim^ NK cells than the HPV (−) CIN 0 group (*P* = 0.026) (Fig. 3h). There were no differences in other NK cell inhibitory phenotypic markers (NKG2A, CD300a LAIR1, and Siglec-7) between the HPV16(+) CIN groups and the HPV(−) CIN 0 group (Fig. 3b–f, i–l) (Table 1). Our study successfully screened TIGIT and KLRG1 from six targets that have always drawn widespread attention, suggesting a possible association between the increased TIGIT and KLRG1 expression on CD56^bright^ NK cells, persistent HPV16 infection and the development of CIN.

### Increased expression of PVR, N-Cadherin and E-Cadherin in HPV16(+)CIN

**Fig. 4:**
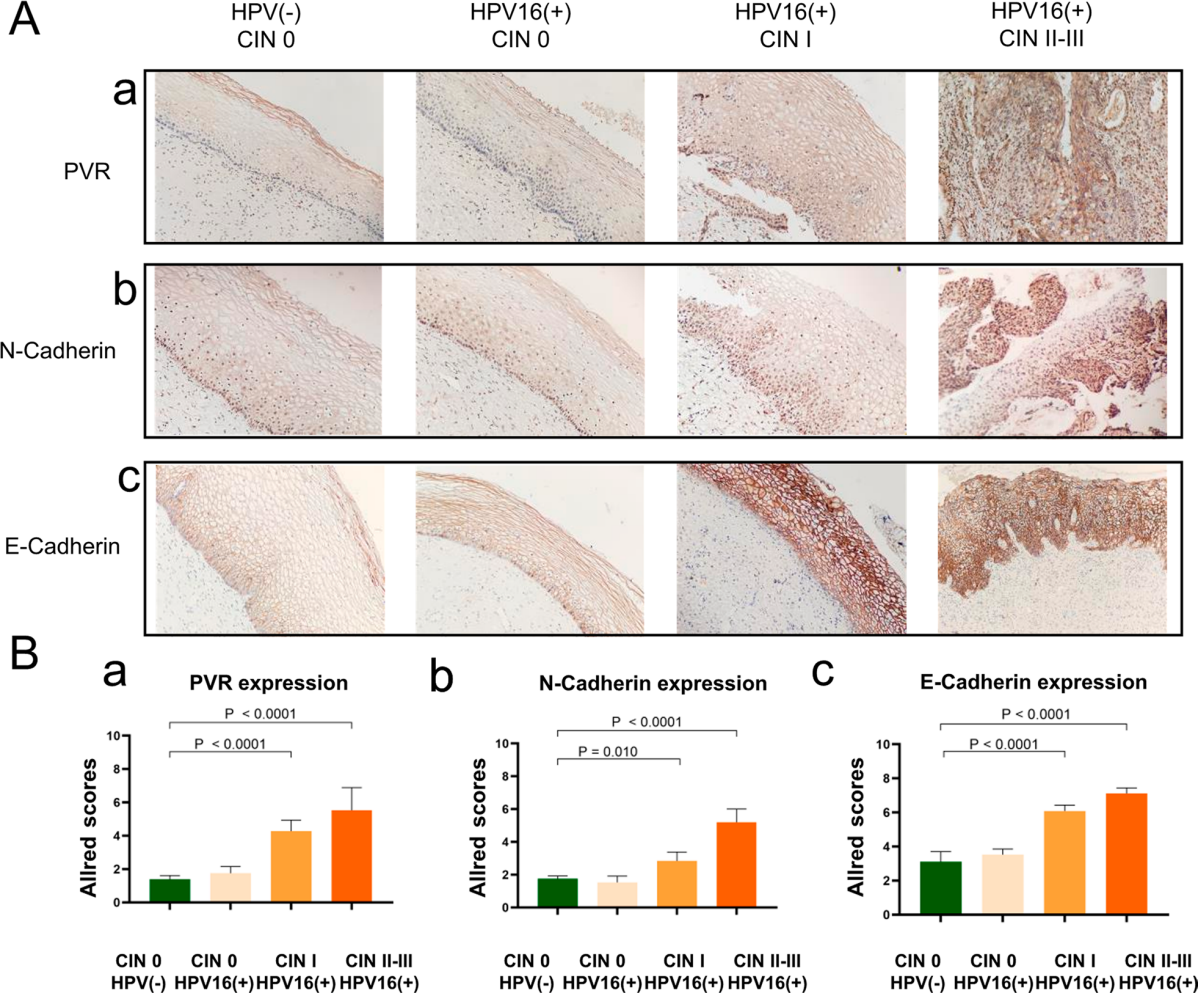
Increased expression of PVR, N-Cadherin and E-Cadherin in HPV16(+) CIN. **A**(a–c) Representative immunohistochemical sections of PVR, E-Cadherin and N-Cadherin expression in cervical tissues with HPV(−) CIN 0 and HPV16(+) CIN. **B**(a, b) Summary of the Allred scores in each group. Error bars indicate SD, original magnification: × 200

We next detected the expression level of PVR (the ligand of TIGIT) and E-Cadherin and N-Cadherin (the ligands of KLRG1) in cervical tissues by immunohistochemical staining. As a member of the Nectin-like protein family, PVR exhibited minor expression in the epithelial layer and stroma of normal cervical tissues. However, comparing with control, an increased expression of PVR in the HPV16(+) groups, particularly in the CIN II–III and CIN I group, was significant (*P* < 0.0001), and the PVR expression level raised with increasing CIN grade (Fig. 4Aa, 4Ba). E-Cadherin and N-Cadherin are known to be important for maintaining membrane integrity and cell function. They were normally expressed on the cell membrane of the epithelial layer, and no difference was observed in the expression intensity between the normal HPV16(+) CIN0 and the control group. However, the expression levels of HPV16(+) CIN I group and CIN II–III group were significantly higher than that of the control group (*P* < 0.0001), and the expression of E-Cadherin and N-Cadherin increased with the increasing CIN grade (Fig. 4A b, c, Bb, c). Hence, the increased PVR, E-Cadherin and N-Cadherin expression on cervical issues suggests that they are involved in the pathological process of HPV16(+) CIN.

## Discussion

Persistent high-risk HPV infection is the most important risk factor of cervical cancer, and the virus clearance barrier caused by immune escape is a key link in the persistent infection of HPV [23]. Prompt detection and clearance of HPV by the acquired immune system are rarely attained, as HPV is an epitheliotropic virus that often establishes local infections. NK cells are incapable of preventing persistent HR-HPV infections among specific populations despite exhibiting effective antiviral activity during the early stage of viral infection. Thus, an in-depth investigation of the evasion mechanism from NK cell-mediated immune responses by HPV is of great clinical significance for the development of therapeutic vaccines and drugs, the elimination of persistent HPV infection, and the effective prevention of cervical cancer [24].

In this study, we investigated the expression of inhibitory receptors and secretion levels of IFN-γ and TNF-α in CD56^bright^ and CD56^dim^ NK cells from HPV(−) women or HPV16(+) women with different CIN grades. The results revealed that the HPV16(+) CIN groups had a significantly increased number of CD56^bright^ NK cells and significantly reduced ability to secrete IFN-γ. Further, the expression of the inhibitory receptors TIGIT and KLRG1, as well as their ligands PVR, N-Cadherin and E-Cadherin were significantly increased. It would be speculated that TIGIT, KLRG1 and its ligands PVR, E-cadherin and N-cadherin are involved in the immune response of CD56^bright^NK cells in HPV16(+) CIN, affecting the immune status of patients in different clinical stages to a certain extent, and promoting the progression of CIN.

NK cells play a key role in the early control of viral infections and tumor immunosurveillance [25, 26]. CD56^dim^ NK cells, which account for approximately 90% of circulating NK cells, carry high levels of perforin and granzyme in the cytoplasm and primarily exert cytotoxic effects [27]. CD56^bright^ NK cells, which account for about 10% of circulating NK cells and are widely present in the human liver and uterus, secrete high levels of cytokines and are key components of the first line of host defense against viral infections. Previous studies have shown that CD56^bright^ NK cells perform important functions in the clearance of HPV-, hepatitis B virus-, and respiratory syncytial virus-infected cells in the early stages, as well as in the prevention of persistent viral infections [28–31]. In some diseases, the CD56^bright^ NK cell subset may be selectively expanded due to its unique cytokine profile, which is consistent with our findings [32]. Our study showed that subjects in the HPV16(+) CIN II–III group had a significantly increased number of circulating CD56^bright^ NK cells. Similarly, the number of circulating NK cells increased, with significantly elevated secretion levels of IFN-γ and TNF-α in the early stage of acute HBV infection. However, different ligands and cytokines render NK cells tolerant to HBV and reduce their antiviral capacity following the progression to chronic infection [33, 34].

In this regard, we speculated that the infection of HPV may locally inhibit the immunomodulatory activity of CD56^bright^ NK cells via certain mechanisms, leading to the compensatory proliferation of this particular NK cell subpopulation. Our subsequent analysis of the ability of the circulating NK cells to secrete effector cytokines showed that CD56^bright^ and CD56^dim^ NK cells in healthy subjects exhibited a remarkable ability to secrete IFN-γ and TNF-α, respectively. However, NK cells, particularly CD56^bright^ NK cells, in subjects with CIN exhibited a significantly reduced ability to secrete IFN-γ, which is consistent with previous findings [35, 36]. Taken together, these findings indicate that the effector function of CD56^bright^ NK cells is reduced during the progression of HPV16(+) CIN, and that the increased number of CD56^bright^ NK cells did not contribute to the timely clearance of virus-infected cells.

NK cell activation is balanced by signals transduced by activating or inhibitory receptors [37]. Under normal conditions, activation relies heavily on the inhibitory receptor to recognize MHC-I molecules to distinguish “self” from “non-self”. Decreased expression or structural abnormalities in MHC-I molecules on the surface of virus-infected cells and tumor cells affect the recognition of ligands by corresponding inhibitory receptors on NK cells, thereby activating NK cells. HPV-mediated immune evasion of CIN and cervical cancer is often accompanied by downregulation of the MHC-I molecules and NK cells would be activated to mediate HPV clearance in this case [14]. However, findings from previous studies have revealed varying degrees of reduction in NK cells function from subjects with HPV-mediated CIN which suggests the involvement of alternative mechanisms to prevent NK cell activation. Some studies have reported that during infections with down-regulated expression of MHC-I molecules, non MHC-I specific inhibitory receptors participate in virus immune escape [38].

Studies have shown that NK cells express some important non-MHC-I inhibitory receptors, such as TIGIT, KLRG1 et al. [19, 39–44]. It is worth pointing out that CD56^bright^ NK cells also highly express a non-canonical MHC-I receptor NKG2A. In order to identify the inhibitory receptors that affect the function of CD56^bright^NK cells in the progression of HPV16(+) CIN, we screened some of candidates recrptor: TIGIT, KLRG1, Siglec-7, LAIR-1, CD300a and NKG2A, and all data were shown in Table 1. Siglec-7 is a member of the immunoglobulin-type lectin (Siglec) family and is highly expressed on NK cells. The ligand of Siglec-7 is sialylated glycans. Studies have shown that NK cell-mediated cytotoxicity was enhanced by desialylation and blockade of Siglec-7 in renal cell carcinoma, melanoma, colon adenocarcinoma, cervical cancer, and chronic myeloid leukemia [19, 39]. NKG2A recognizes the non-classical MHC-I molecule HLA-E, and the expression of NKG2A is found to be up-regulated in peripheral blood NK cells of patients with chronic hepatitis. Antiviral therapy against NKG2A in patients with chronic hepatitis causes NK cells to produce more IFN-γ [40]. At present, humanized anti-NKG2A monoclonal antibody (IPH2201, monalizumab) has been used in several clinical trials [41]. Leukocyte-associated immunoglobulin-like receptor-1 (LAIR-1) widely exists in immune cells, and the ligand of LAIR-1 is collagen, indicating that extracellular matrix may have the function of immune regulation. Relevant studies have shown that LAIR-1 can mediate immune escape in oral squamous cell carcinoma and hepatocellular carcinoma [42, 43]. Phosphatidylserine (PS)/phosphatidylethanolamine (PE) interacts with CD300a, revealing its role in immune function regulation and involvement in disease host responses. CD300a exhibits therapeutic potential in HIV infection, CMV and Pichinde virus infection [44]. However, our screening results showed that Siglec-7, NKG2A, LAIR-1 and CD300a were not significantly different in our group. These receptors may be less responsive in the early stages of chronic HPV16 infection. Alternatively, the progression from CIN to cervical cancer takes a certain amount of time, and the pathological changes of the two disease states are quite different. Our future studies will further explore the expression of these receptors in cervical cancer.

Our results confirmed an increased proportion of TIGIT expressed on CD56^bright^ NK cells in HPV16(+) CIN. Although there was no statistically significant, the MFI of TIGIT showed a consistent trend with percentage across groups. We consider that persistent HPV infection may preferentially stimulate the overall increase in the proportion of TIGIT expressed on CD56^bright^ NK cells, and individual differences between samples may also be the reason for this phenomenon. Previous studies have shown that TIGIT is involved in the immune response to tumor and viral infections. Johnston et al. [45] had previously demonstrated that TIGIT was significantly upregulated in tumor-infiltrating lymphocytes in endometriosis, breast cancer, clear cell renal cell carcinoma, non-small cell lung cancer, and colorectal cancer. In addition, patients infected with human immunodeficiency virus (HIV) had a higher TIGIT expression level in NK cells than HIV. In this study, researchers found that high levels of TIGIT reversibly inhibited IFN-γ production in the NK cells [46]. Previous studies have indicated that the inhibitory interaction of the TIGIT-PVR axis is critical for the functional heterogeneity of NK cells, by which it negatively regulates cell viability and induces immune tolerance [33, 34]. We observed that PVR, the ligand of TIGIT, had a relatively high expression level in HPV16(+) subjects with CIN. Therefore, we speculated that PVR might respond to TIGIT, although we do not have sufficient evidence to prove that TIGIT-PVR is involved in HPV16 immune evasion. Our results could hypothesis that the TIGIT-PVR pathway may be an important negative regulator for inducing HPV16 immune evasion, which will be our important research work in the future. In fact, clinical trials of anti-TIGIT antibodies are ongoing. For instance, the Phase II CITYSCAPE trial evaluated the combination of tiragolumab (anti-TIGIT antibody) and atezolizumab (anti-PD-L1 antibody) in the treatment of non-small cell lung cancer with encouraging results [47].

KLRG1 is the other important non-MHC-I specific receptor on NK cells. Cadherin type I, such as -E, N cadherin, has been identified as the ligand of KLRG1 [48]. KLRG1 is a transmembrane receptor of the lectin-like superfamily predominantly mediating inhibitory effect through the cytoplasmic ITIM motif. Previous studies showed that KLRG1 was rapidly upregulated on NK cells, CD8^+^ and CD4^+^ T cells during chronic infections with the human cytomegalovirus, Epstein-Barr virus, and HIV [48, 49]. Additionally, it was found that the expression of KLRG1 increased in chronic hepatitis C [50]. Ito et al. [49] showed that the interaction of cadherin with KLRG1 activated the inhibitory pathway of NK cells. Our results showed the expression of KLRG1 on circulating CD56^bright^ NK cells was increased primarily in HPV16(+) subjects with CIN 0 and CIN I. Although its characteristic expression in peripheral blood circulation was not found in high-grade CIN. It has been reported that KLRG1 is widely used as a differentiation marker, and the expression of KLRG1 increases with differentiation. The highest percentage of KLRG1 expression is observed on highly differentiated end-stage cells [51], which may support our experimental results. We observed that HPV16(+) CD56^bright^NK cells in CIN II–III displayed abnormal increase in number and decreased function, implying more poorly differentiation of CD56^bright^NK cells compared to HPV16(+) low-grade group. Cervical histochemical results showed that the expression of its ligands E, N-cadherin in CIN II–III was significantly increased, and E-Cadherin was also increased in CIN I. It may be noted that even though the expression of E-cadherin is reduced in most tumor studies to increase cell invasiveness, correlation studies showed that some tumors may paradoxically use KLRG1-cadherin to evade immune surveillance. For example, in invasive breast cancer, E-cadherin was down-regulated initially by some tumor cells to acquire metastatic potential, and then it was re-expressed to promote adhesion and evade immune attack [52]. It is still noteworthy that the KLRG1 expression on circulating CD56^dim^ NK cells was increased significantly, which needs to be investigated in future experiments to clarify the effect of CD56^dim^ NK cells on HPV16(+) CIN.

## Conclusion

In this study, we have demonstrated a correlation between upregulated expression of TIGIT, KLRG1 and PVR, E-cadherin and N-cadherin on CD56^bright^NK cells and increased CIN grade in cervical in situ tissue in HPV16 infection. The up-regulated TIGIT-PVR and KLRG1-E-cadherin/N-cadherin pathways may contribute, at least in part, to CD56^bright^NK cell functional impairments, which in turn promotes HPV16-associated CIN progression. Therefore, increased numbers of TIGIT^+^CD56^bright^ NK and KLRG1^+^CD56^bright^ NK cells may serve as promising early markers of HPV16-associated CIN progression, and we highly expect that TIGIT- and KLRG1-related immunotherapies will bring hope to patients with HPV16-associated CIN.

## Data Availability

Not applicable.

## Abbreviations

CIN: Cervical intraepithelial neoplasia
HE: Hematoxylin–eosin
HR-HPV: High-risk human papillomavirus
IHC: Immunohistochemistry
IFN-γ: Interferon-γ
KLRG1: Killer cell lectin-like receptor G1
LAIR-1: Leukocyte-associated Ig-like receptor-1
MHC: Major histocompatibility complex
MFI: Median fluorescence intensity
NK: Natural killer cells
PBMC: Peripheral blood mononuclear cell
PVR: Poliovirus receptor
Siglec-7: Sialic acid-binding immunoglobulin-like lectins 7
TIGIT: T cell immunoglobulin and immunoreceptor tyrosine-based inhibitory motif (ITIM) domain
TNF-α: Tumor necrosis factor-α
